# Integrated Characterization of *Wolffia globosa* as a Potential Food Ingredient: Nutritional and Physicochemical Properties, Selected Elements, and Preliminary In Vitro Responses

**DOI:** 10.3390/foods15162919

**Published:** 2026-08-20

**Authors:** Anusorn Tongyai, Veeradej Pisprasert, Kukiat Tudpor, Nitchara Toontom

**Affiliations:** 1 Faculty of Public Health, Mahasarakham University, Maha Sarakham 44150, Thailand; anusorn1408@gmail.com; 2 Division of Clinical Nutrition, Department of Medicine, Faculty of Medicine, Khon Kaen University, Khon Kaen 40002, Thailand; pveera@kku.ac.th; 3 Public Health and Environmental Policy in Southeast Asia Research Cluster (PHEP-SEA), Faculty of Public Health, Mahasarakham University, Maha Sarakham 44150, Thailand; kukiat.t@msu.ac.th

**Keywords:** *Wolffia globosa*, watermeal, nutritional composition, plant-based food ingredient, physicochemical properties, selected elements, DPPH radical-scavenging activity, IL-6 production, cell viability

## Abstract

*Wolffia globosa* (watermeal) is a potential plant-based food ingredient. This study characterized its nutritional and physicochemical properties, elemental levels, total phenolic content (TPC), total flavonoid content (TFC), DPPH radical-scavenging activity, IL-6 production in H_2_O_2_-stimulated RAW264.7 macrophages, and preliminary cell-viability responses. On a dry-weight basis, the tested *W. globosa* sample contained 25.83% protein, 4.56% fat, 16.58% ash, 8.80% crude fiber, and 44.22% carbohydrate. Arsenic and zinc were detected below their limits of quantification, whereas cadmium, copper, and lead were below their method detection limits. All analytical reporting limits were below the corresponding EFSA specification values. The extract had TPC and TFC values of 0.85 ± 0.03 mg GAE/g sample DW and 0.52 ± 0.02 mg QE/g sample DW, respectively, and showed weak DPPH radical-scavenging activity (IC_50_ = 7.58 ± 0.32 mg DW-equivalent/mL). At 0.1 mg DW-equivalent/mL, the H_2_O_2_-stimulated extract-treated group showed a lower IL-6 concentration than the H_2_O_2_-stimulated extract-free model control. Weak reductions in cancer-cell viability occurred only at high concentrations (IC_50_ values of approximately 38–42 mg DW-equivalent/mL). The biological findings remain preliminary. Overall, the tested sample showed promising nutritional and physicochemical characteristics, low selected-elements levels, and preliminary in vitro responses.

## 1. Introduction

*Wolffia globosa* (Roxb.) Hartog & Plas is a species within the *Lemnaceae* (duckweed) family, commonly known as watermeal and locally referred to as “kai phum” in Thailand. It has gained increasing attention as a potential food resource because of the growing demand for plant-based protein and sustainable food alternatives, as well as its capacity for rapid biomass production under suitable cultivation conditions. Recent studies have shown that *W. globosa* is nutrient-dense, with high protein content, appreciable dietary fiber, and key micronutrients, supporting its potential use as a functional ingredient and an alternative plant-based protein source for food and health applications [1,2,3]. However, given its extremely high moisture content in the fresh state, *W. globosa* is more appropriately used in processed forms, such as dried biomass or powdered ingredients, rather than fresh. Therefore, its nutritional and functional properties are more meaningfully interpreted in the context of concentrated or processed applications.

In addition to its nutritional value, *W. globosa* contains bioactive constituents including phenolic compounds, flavonoids, and carotenoid-related pigments, which are associated with antioxidant activity. A recent food application study of *W. globosa* powder reported increased total phenolic and flavonoid content, as well as antioxidant capacity, in fortified snack formulations. At the same time, broader reviews of duckweed species, including *W. globosa*, have also highlighted phytochemicals such as lutein and β-carotene as important contributors to the biological value of this plant group [2,3]. Accordingly, the antioxidant potential of *W. globosa* is biologically plausible based on its reported phytochemical composition, although further extract-level evaluation is required to clarify its functional activity. Beyond antioxidant activity, recent evidence also supports the anti-inflammatory potential of *W. globosa*-derived extracts. In a 2025 study, protein extracts from dried *W. globosa* reduced the secretion of pro-inflammatory cytokines, including IL-1β and IL-6, in THP-1-derived macrophage-like cells, accompanied by downregulation of inflammation-related signaling proteins [4]. These findings suggest that *W. globosa* may contain compounds that modulate inflammatory responses. However, identifying specific bioactive constituents and underlying mechanisms is beyond the scope of the present extract-level screening study.

Previous work has reported inhibitory effects of protein extracts and enzymatically modified protein preparations from *W. globosa* in selected cancer cell models [5]. However, those findings were obtained using protein-enriched preparations and should not be extrapolated to crude whole-plant extracts or interpreted as evidence of anticancer efficacy. Evaluation of crude extracts in cancer cell lines may nevertheless provide preliminary information on concentration-dependent changes in cell viability.

Recent cultivation and food-development studies on duckweed species, including *W. globosa*, have highlighted its potential for food applications while also showing that composition can vary with production and processing conditions [6].

Previous studies have provided comprehensive information on the nutritional composition, functional properties, and selected biological characteristics of *Wolffia globosa* [1], while other investigations have focused on its incorporation into food products [2,6] or on the biological activities of protein, peptide, and other extracted fractions [4,5,7,8]. Thus, the novelty of the present study does not lie in any individual analytical method. Rather, its contribution is the coordinated assessment of proximate composition, physicochemical properties, selected elemental levels, phytochemical content, and preliminary in vitro responses, using biomass and ethanolic extract prepared from the same study material and following the same processing sequence. This common experimental basis allows the compositional and preliminary biological findings to be interpreted within a defined sample context, rather than being inferred solely from separate studies using different cultivation sources, processing conditions, or extract preparations.

Accordingly, the present study provides a consolidated baseline characterization of the tested *W. globosa* material for subsequent research on ingredient processing, food formulation, and biological evaluation. The study was not designed to establish compound-specific mechanisms or to generalize the findings across cultivation sources and processing conditions.

Therefore, the present study aimed to characterize the nutritional and physicochemical properties, selected elemental levels, total phenolic and total flavonoid contents, and DPPH radical-scavenging activity of *W. globosa* and to evaluate the effects of its extract on IL-6 production in H_2_O_2_-stimulated RAW264.7 macrophages and on cell viability. The study was designed to provide coordinated baseline data relevant to the further evaluation of the tested material as a potential plant-based food ingredient.

## 2. Materials and Methods

### 2.1. Chemicals and Reagents

All chemicals and reagents used in this study were of analytical grade or higher. Ethanol (HPLC grade) and petroleum ether (boiling range 40–60 °C) were obtained from Merck (Darmstadt, Germany). Folin–Ciocalteu reagent, sodium carbonate, aluminum chloride hexahydrate (AlCl_3_·6H_2_O), gallic acid, quercetin, and 2,2-diphenyl-1-picrylhydrazyl (DPPH) were purchased from Sigma-Aldrich (St. Louis, MO, USA). Nitric acid (HNO_3_, analytical grade), hydrogen peroxide (H_2_O_2_, 30%), and dimethyl sulfoxide (DMSO) were also obtained from Merck (Darmstadt, Germany). Multi-element standard solutions for inductively coupled plasma mass spectrometry (ICP-MS) calibration were purchased from Inorganic Ventures (Christiansburg, VA, USA). Cell culture media, including Dulbecco’s Modified Eagle’s Medium (DMEM), fetal bovine serum (FBS), and antibiotics (penicillin–streptomycin), were obtained from Gibco (Thermo Fisher Scientific, Waltham, MA, USA). A Mouse IL-6 SimpleStep ELISA^®^ Kit (ab222503, Abcam, Cambridge, UK) was used to determine IL-6 levels. All cell culture reagents and assay kits were handled under sterile conditions.

### 2.2. Sample Preparation and Extraction of W. globosa

Fresh *Wolffia globosa* samples were obtained from a single local cultivation source in Phra Nakhon Si Ayutthaya, Thailand, and were collected as a single pooled batch. Species identification was based on morphological characteristics consistent with *W. globosa* (Roxb.) Hartog & Plas; no voucher specimen was deposited. Detailed records of the harvest date, cultivation conditions, water quality, nutrient management, and maturity stage were not available from the cultivation source. The samples were thoroughly washed with distilled water to remove surface impurities. Immediately after washing, a separate aliquot of the fresh, unboiled material was reserved for moisture content and water activity measurements. The remaining material was boiled in distilled water at a 1:10 (*w*/*v*) sample-to-water ratio for 15 min. The boiled material was then drained, rapidly frozen at −76 °C, and stored until further processing.

The frozen, boiled material was freeze-dried and ground into a fine powder using a laboratory grinder. The powder was passed through a 60-mesh sieve and stored in airtight containers at −20 °C until analysis. Unless otherwise stated, proximate-composition, physicochemical, selected-element, and extract-based analyses were conducted using this boiled and freeze-dried powder. Only moisture content and water activity were measured using the fresh, washed, unboiled aliquot.

For extraction, 193.2 g of freeze-dried *W. globosa* powder obtained from the pooled sample batch was extracted with 70% (*v*/*v*) aqueous ethanol at a solid-to-liquid ratio of 1:30 (*w*/*v*), using approximately 5.8 L of extraction solvent. The extraction conditions were adopted from a previously reported procedure for *W. globosa* [2] to prepare a crude extract for preliminary phytochemical and in vitro evaluation. The mixture was shaken in a water-bath shaker (Memmert GmbH + Co. KG, Schwabach, Germany) for 2 h at 50 °C and centrifuged at 3800× *g* for 10 min using a refrigerated centrifuge (Hettich^®^ ROTINA 38R, Andreas Hettich GmbH, Tuttlingen, Germany).

Before the cell-based assays, the ethanol-containing supernatant was concentrated under reduced pressure at 45 °C using a rotary evaporator to a final liquid volume of 24.5 mL. The post-evaporation liquid concentrate was stored at −20 °C until analysis. The concentrate was not dried or weighed; therefore, a dry-mass extraction yield, expressed as the mass of dried extract relative to the starting material, could not be determined. The final volume of 24.5 mL represents the volume of the liquid concentrate and should not be interpreted as an extraction yield.

For concentration reporting, the liquid concentrate was assigned a nominal starting-material-equivalent concentration of 7885.7 mg DW-equivalent/mL, calculated from 193,200 mg of starting freeze-dried material divided by the final concentrate volume of 24.5 mL. Accordingly, the treatment concentrations reported in this study represent the equivalent mass of the starting freeze-dried material per milliliter of final assay medium rather than the mass of dried extract solids. Serial dilutions were prepared directly in DMEM without adding ethanol or any other carrier solvent. Residual ethanol in the post-evaporation concentrate was not analytically quantified, and concentration-matched ethanol vehicle controls were not included. Across the tested range of 0.001–50 mg DW-equivalent/mL, the post-evaporation liquid concentrate constituted approximately 0.0000127–0.634% (*v*/*v*) of the final assay medium.

Chemical characterization of the extract was limited to the spectrophotometric determination of total phenolic and total flavonoid contents. No chromatographic or spectroscopic identification of individual compounds was performed in this study.

All analyses were conducted using material derived from the same pooled sample batch. Triplicate measurements in the compositional and physicochemical analyses were technical replicate measurements of this pooled material. The phytochemical and cell-based assays were conducted using a single extraction batch, with measurements performed in triplicate as technical assay replicates. The study did not include independent biological replicates from separate cultivation or harvest batches or independent extraction batches.

### 2.3. Proximate Composition Analysis

The proximate composition of the boiled and freeze-dried *W. globosa* powder was determined using AOAC-based methods selected according to the sample matrix and the analytical principle of each method [9]. Moisture content was determined separately using the fresh, washed, unboiled aliquot by oven-drying at 105 °C to a constant mass, using AOAC Method 930.04 as the reference method for the plant matrix.

Crude protein was determined by the Kjeldahl method using a Kjeltec 2300 Analyzer Unit (FOSS, Hillerød, Denmark), according to AOAC Method 978.04. Protein content was calculated from the measured nitrogen content using a nitrogen-to-protein conversion factor of 6.25. Crude fat was determined by acid hydrolysis followed by petroleum ether extraction using a Hydrotec™ 8000 unit and Soxtec™ 8000 system (FOSS, Hillerød, Denmark), using an automated adaptation of AOAC Method 922.06. Ash content was determined by incineration in a muffle furnace at 550 °C for 6 h according to AOAC Method 930.05. Crude fiber was determined using a FiberCap system (FOSS, Hillerød, Denmark) according to the Ceramic Fiber Filter Method described in AOAC Method 962.09. The measured fiber fraction is therefore reported as crude fiber and should not be interpreted as dietary fiber.

Carbohydrate content was calculated by difference on a dry-weight basis as follows:

Carbohydrate (% DW) = 100 − [Protein (% DW) + Fat (% DW) + Ash (% DW) + Crude fiber (% DW)].

Energy content was calculated on a dry-weight basis using the Atwater factors as follows:

Energy (kcal/100 g DW) = [Protein (g/100 g DW) × 4] + [Fat (g/100 g DW) × 9] + [Carbohydrate by difference (g/100 g DW) × 4].

Crude fiber was reported separately and was not assigned an additional energy factor. Each reported compositional value was based on three technical replicate measurements of material obtained from the same pooled sample batch.

Water activity (a_w_) was measured using the same fresh, washed, unboiled aliquot employed for moisture determination. Approximately 5 g of sample was placed in the sample cup of a LabSwift-aw water activity meter (Novasina AG, Lachen, Switzerland) and measured at 25 ± 1 °C. The sample was equilibrated until two consecutive readings differed by less than 0.001 a_w_ units. The measurement followed the instrumental principle described in AOAC Method 978.18 [10].

### 2.4. Physicochemical Analysis

Physicochemical properties of *W. globosa* powder were evaluated by measuring color parameters, pH, and fat-binding capacity (FBC), as described below.

#### 2.4.1. Color Parameters

Color parameters of *W. globosa* powder were measured using a ColorFlex EZ colorimeter (HunterLab, Reston, VA, USA). The instrument was calibrated using a standard white tile according to the manufacturer’s instructions before analysis. Color was expressed in the CIE L* (lightness), a* (redness/greenness), and b* (yellowness/blueness) system. Measurements were performed in triplicate, and the results were reported as the mean ± standard deviation (SD), following standard colorimetric evaluation methods [11].

#### 2.4.2. pH

The pH of *W. globosa* was measured using a digital pH meter (SevenCompact™, Mettler Toledo, Greifensee, Switzerland). The instrument was calibrated with standard buffer solutions at pH 4.0, 7.0, and 10.0 before use. The sample was dispersed in distilled water at a 1:10 (*w*/*v*) ratio prior to measurement, and pH values were recorded in triplicate and expressed as the mean ± SD [10].

#### 2.4.3. Fat-Binding Capacity

Fat-binding capacity (FBC) was determined using a gravimetric centrifugation method with minor modifications [7]. Briefly, 4 g of the sample was mixed with 20 mL of maize oil in a 50 mL centrifuge tube and manually shaken intermittently for 30 min. The mixture was then centrifuged at 1600× *g* for 25 min, and the unbound oil was decanted. The amount of oil retained by the sample was determined gravimetrically and expressed as mL oil/g sample.

### 2.5. Total Phenolic and Total Flavonoid Content Assays

Total phenolic and total flavonoid contents were expressed relative to the initial freeze-dried sample weight as mg GAE/g DW of sample and mg QE/g DW of sample, respectively. The post-evaporation liquid concentrate described in Section 2.2 was used for these assays. Because the concentrate was not dried and weighed, the reported values are expressed relative to the dry weight of the starting sample and do not represent per-gram values for the dried extract.

#### 2.5.1. Total Phenolic Content (TPC)

Total phenolic content was determined using the Folin–Ciocalteu colorimetric method based on the procedure of Bozin et al. [12], with minor modifications. Briefly, 500 μL of extract was mixed with Folin–Ciocalteu reagent at an extract-to-reagent ratio of 1:5 (*v*/*v*), followed by the addition of 7.5% (*w*/*v*) sodium carbonate solution. The mixture was incubated at room temperature in the dark for 30 min, and absorbance was measured at 760 nm using a UV–Visible spectrophotometer (Shimadzu UV-1800, Kyoto, Japan). Gallic acid was used to prepare the calibration curve, and the results were expressed as mg gallic acid equivalents per gram of sample dry weight (mg GAE/g DW). All analyses were performed in triplicate, and the results were reported as mean ± SD.

#### 2.5.2. Total Flavonoid Content (TFC)

Total flavonoid content was determined using the aluminum chloride colorimetric method as described by Bozin et al. [12], with minor modifications. Briefly, 1 mL of diluted extract was mixed with 1 mL of 2% AlCl_3_·6H_2_O solution and incubated at room temperature for 15 min. Absorbance was measured at 430 nm using a UV–Visible spectrophotometer (Shimadzu UV-1800, Kyoto, Japan). Quercetin was used to construct the calibration curve, and the results were expressed as mg quercetin equivalents per gram of sample dry weight (mg QE/g DW of sample). All analyses were performed in triplicate, and the results were reported as mean ± SD.

#### 2.5.3. DPPH Radical-Scavenging Activity Assay

DPPH radical-scavenging activity was evaluated as described by Brand-Williams et al. [13], with minor modifications. A 100 μM DPPH solution was prepared in ethanol. Briefly, 200 μL of extract at different concentrations was added to a 96-well microplate, followed by 100 μL of DPPH solution. The reaction mixture was incubated at room temperature in the dark for 30 min, and absorbance was measured at 515 nm using a UV–Visible spectrophotometer (Shimadzu UV-1800, Kyoto, Japan). Radical-scavenging activity was calculated as follows:

DPPH scavenging activity (%) = [(*A*_control_ − *A*_sample_)/*A*_control_] × 100

where *A*_control_ is the absorbance of the control and *A*_sample_ is the absorbance of the extract-containing reaction mixture. IC_50_ values were determined from dose–response curves and expressed as mg DW-equivalent/mL, where DW-equivalent denotes the equivalent mass of the starting freeze-dried *W. globosa* material rather than the mass of dried extract solids. The DPPH assay was used as a preliminary measure of the extract’s radical-scavenging activity. All measurements were performed in triplicate and expressed as mean ± SD.

### 2.6. Selected Elemental Analysis

Dried *W. globosa* samples (approximately 0.5 g) were ground to a fine powder and digested with concentrated nitric acid and hydrogen peroxide in a closed-vessel microwave digestion system, with a sample-to-acid ratio of approximately 1:20 (*w*/*v*), until a clear solution was obtained. The digest was filtered and diluted to volume with deionized water before analysis. Arsenic (As), cadmium (Cd), copper (Cu), zinc (Zn), and lead (Pb) were quantified by inductively coupled plasma mass spectrometry (ICP–MS; NexION 2000, PerkinElmer, Waltham, MA, USA). Calibration was performed using multi-element standard solutions (Inorganic Ventures, Christiansburg, VA, USA), and analytical blanks and a quality-control reference standard (Multi-Element Calibration Standard 3, PerkinElmer, Waltham, MA, USA; Part No. N9300233) were included for quality control.

Method performance was assessed using the quality-control reference standard, with recoveries ranging from 76% to 109% for the elements analyzed. The method detection limits (MDLs) were 0.010, 0.010, 0.100, 0.320, and 0.040 mg/kg DW for As, Cd, Cu, Zn, and Pb, respectively. The corresponding limits of quantification (LOQs) were 0.050, 0.050, 0.500, 0.500, and 0.100 mg/kg DW, respectively. Results below the MDL were reported as <MDL, whereas signals at or above the MDL but below the LOQ were reported as detected, <LOQ, and were not interpreted as quantitative concentrations. Measurements were performed as three technical replicate measurements of material from the same pooled sample batch.

### 2.7. Cell Viability Assay in Cancer Cell Lines

The effects of *W. globosa* extract on the viability of cancer cell lines were evaluated using the MTT assay as described by Gorantla et al. [14]. HepG2 (human hepatocellular carcinoma), A549 (human lung adenocarcinoma), and Caco-2 (human colorectal adenocarcinoma) cells were seeded in 96-well plates at a density of 7 × 10^3^ cells/well and cultured for 24 h at 37 °C in a humidified atmosphere containing 5% CO_2_. Cells were maintained in DMEM supplemented with 10% FBS and 1% penicillin–streptomycin. After incubation, cells were treated with serial dilutions of the post-evaporation liquid concentrate prepared in DMEM at final concentrations of 0.001, 0.01, 0.1, 1, 10, and 50 mg DW-equivalent/mL (100 μL/well) for 24 h. Following treatment, 100 μL of MTT solution (0.5 mg/mL) was added to each well, and the plates were incubated for 2 h in the dark. The supernatant was removed, and the resulting formazan crystals were dissolved in 100 μL of DMSO. Absorbance was measured at 570 nm using a microplate reader (BioTek Synergy HT, Winooski, VT, USA). Extract blanks containing the extract at the corresponding concentrations and MTT reagent, but no cells, were included to correct for background absorbance. Cell viability was calculated as follows:

(1) $$Cell\;viability\left(\%\right)=\frac{A_{treated}-A_{blank}}{A_{control}-A_{blank}}\times 100$$

where $A_{\mathrm{treated}}$ is the absorbance of extract-treated cells and $A_{\mathrm{control}}$ is the absorbance of untreated control cells, and $A_{blank}$ is the absorbance of the corresponding extract blank without cells. IC_50_ values were obtained from the dose–response curves and expressed as mg DW-equivalent/mL. The assay was performed in three technical replicates using a single extraction batch, and the results were expressed as the mean ± SD.

### 2.8. IL-6 Production in H_2_O_2_-Stimulated RAW264.7 Macrophages

The effect of *W. globosa* extract on IL-6 production was evaluated in H_2_O_2_-stimulated RAW264.7 murine macrophages using an ELISA-based method. H_2_O_2_ was used as a defined oxidative-stress stimulus to examine the macrophage IL-6 response following oxidative challenge, rather than as a direct substitute for the more conventional LPS-induced inflammatory model [15]. RAW264.7 cells were seeded in 6-well plates at a density of 2 × 10^5^ cells/well and incubated at 37 °C in a humidified atmosphere containing 5% CO_2_. The cells were exposed to 1 mM H_2_O_2_ for 30 min. After the 30 min exposure, the H_2_O_2_-containing medium was removed and replaced with fresh DMEM containing the post-evaporation *W. globosa* concentrate at a final concentration of 0.1 mg DW-equivalent/mL. The cells were then incubated for a further 24 h.

The experimental groups comprised an untreated control, an H_2_O_2_-stimulated extract-free model control, and an H_2_O_2_-stimulated group treated with *W. globosa* extract. The H_2_O_2_-stimulated group served as the model control and was not a pharmacological positive control for anti-inflammatory activity. A concentration-matched vehicle control, an extract-only group within the IL-6 experiment, a recognized pharmacological positive control, and an extract dose–response series were not included. Cell viability was not assessed under the combined H_2_O_2_-plus-extract treatment condition.

After treatment, the culture supernatants were collected and centrifuged at 2000× *g* for 10 min to remove cell debris. IL-6 concentrations were determined using a Mouse IL-6 SimpleStep ELISA^®^ Kit (ab222503, Abcam, Cambridge, UK; detection range 15.6–1000 pg/mL; analytical sensitivity 11.3 pg/mL) according to the manufacturer’s instructions. Absorbance was measured at 450 nm using a microplate reader (BioTek Synergy HT, Winooski, VT, USA), and IL-6 concentrations were calculated from the corresponding standard curve. Measurements were performed in triplicate as technical assay replicates using a single extraction batch, and the results were expressed as the mean ± standard deviation.

### 2.9. Cell Viability Assay in RAW264.7 Macrophages

The effect of *W. globosa extract* on RAW264.7 cell viability was assessed using the MTT assay, as described by Gorantla et al. [14], with minor modifications. RAW264.7 murine macrophages were seeded in 96-well plates at a density of 7 × 10^3^ cells/well in DMEM supplemented with 10% FBS and 1% penicillin–streptomycin and incubated for 24 h at 37 °C in a humidified atmosphere containing 5% CO_2_. Cells were then treated with serial dilutions of the post-evaporation liquid concentrate prepared in DMEM at final concentrations of 0.001, 0.01, 0.1, 1, 10, and 50 mg DW-equivalent/mL (100 μL/well) for 24 h. After treatment, 100 μL of MTT solution (0.5 mg/mL) was added to each well, and the plates were incubated in the dark for 2 h. The supernatant was removed, and the formazan crystals were dissolved in 100 μL of DMSO. Absorbance was measured at 570 nm using a microplate reader (BioTek Synergy HT, Winooski, VT, USA). Extract blanks were included for background correction as described in Section 2.7. Cell viability was calculated using Equation (1). IC_50_ values were estimated from dose–response curves where applicable and expressed as mg DW-equivalent/mL. The assay was performed in three technical replicates using a single extraction batch, and the results were expressed as the mean ± SD.

### 2.10. Statistical Analysis

All reported values with *n* = 3 represent technical replicate measurements rather than independent biological replicates. For compositional, physicochemical, and selected-element analyses, the technical replicates were obtained from the same pooled sample batch, whereas the extract-based assays were performed as technical assay replicates using a single extraction batch. Results were expressed as the mean ± standard deviation (SD). When comparisons among treatment groups or concentrations were made, differences were evaluated using one-way analysis of variance (ANOVA) followed by Duncan’s multiple range test. Differences were considered statistically significant at *p* < 0.05. Because the replicates were technical, these statistical comparisons describe within-assay technical variation and should not be interpreted as estimates of biological variation or reproducibility across independent *W. globosa* samples or batches. Statistical analyses were performed using IBM SPSS Statistics version 19.0 (IBM Corp., Armonk, NY, USA).

## 3. Results

### 3.1. Nutritional Composition

**Figure 1:**
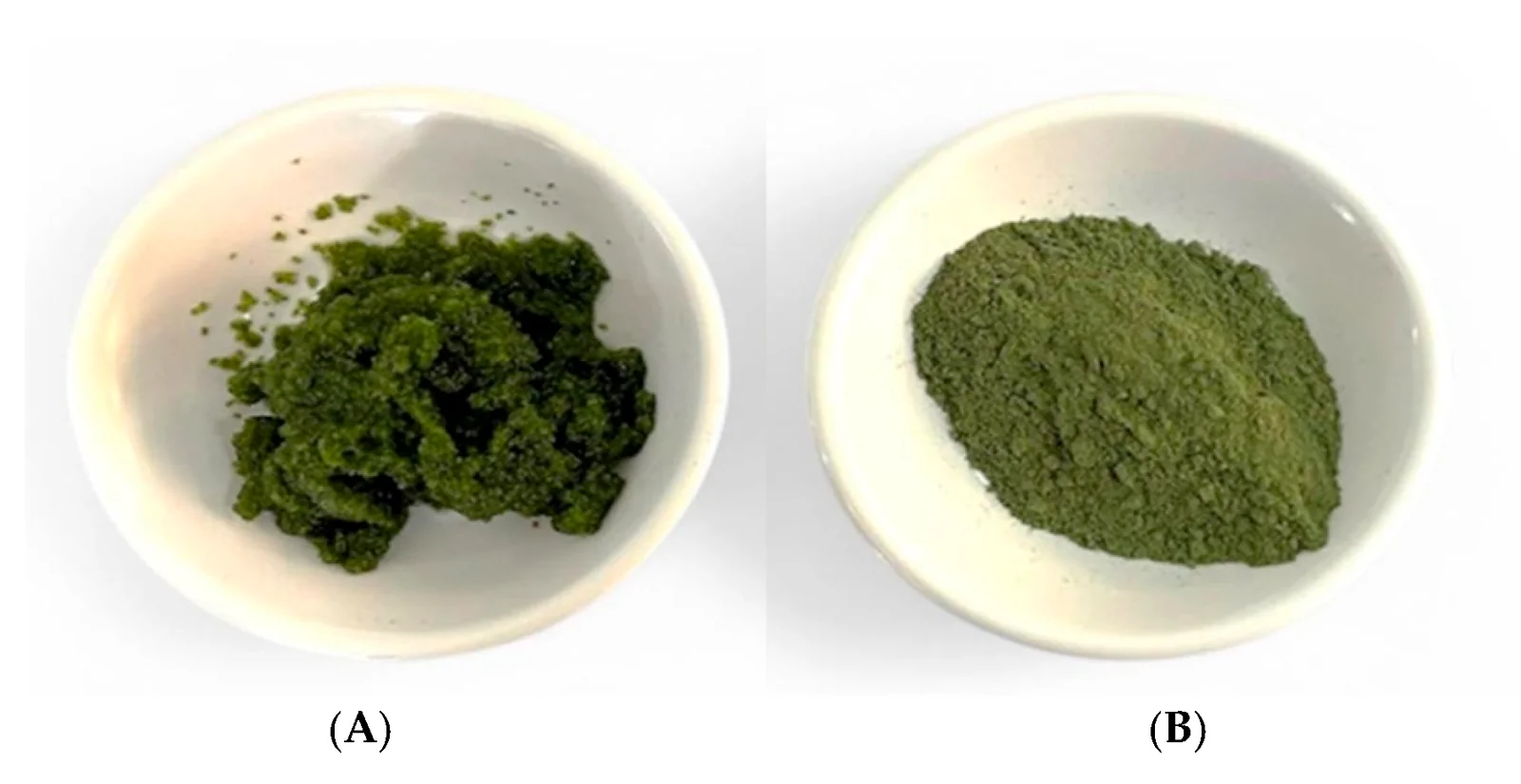
Representative appearance of (**A**) fresh and (**B**) freeze-dried *W. globosa* powder used in this study.

Representative appearances of the fresh and freeze-dried *W. globosa* samples used in this study are shown in Figure 1. The proximate composition of the freeze-dried powder from the tested pooled batch is presented on a dry-weight basis in Table 1. Protein and crude fiber contents were 25.83 ± 2.65% and 8.80 ± 0.61%, respectively, and the calculated energy content was 321.24 ± 1.63 kcal/100 g DW. The measured fiber fraction is reported as crude fiber; dietary fiber was not determined in this study.

Moisture content and water activity were measured separately using fresh material after washing and before boiling. As shown in Table 2, the fresh material contained 96.78 ± 0.07% moisture and had a water activity of 0.96 ± 0.01. Thus, the results in Table 1 and Table 2 represent freeze-dried powder and fresh material, respectively.

### 3.2. Physicochemical Properties

The physicochemical characteristics of the freeze-dried powder from the tested pooled batch are summarized in Table 3. The sample showed negative a* and positive b* values, a near-neutral pH, and measurable fat-binding capacity.

### 3.3. Total Phenolic and Total Flavonoid Contents and DPPH Radical-Scavenging Activity

The total phenolic and total flavonoid contents of the extract prepared from the tested pooled batch are presented in Table 4. The values were 0.85 ± 0.03 mg GAE/g DW of sample and 0.52 ± 0.02 mg QE/g DW of sample, respectively. These values represent equivalent responses obtained from the Folin–Ciocalteu and aluminum chloride colorimetric assays and do not identify or quantify individual phenolic or flavonoid compounds.

The *W. globosa* extract showed weak DPPH radical-scavenging activity, with an IC_50_ value of 7.58 ± 0.32 mg DW-equivalent/mL. This value represents the concentration required to reduce the DPPH radical signal by 50% under the specified assay conditions.

### 3.4. Levels of Selected Elements

The levels of the five selected elements in the freeze-dried powder from the tested pooled batch, relative to the analytical reporting limits, are presented in Table 5. Arsenic and zinc produced analytical signals at or above their respective MDLs but below their LOQs and were therefore reported as detected, <LOQ. Cadmium, copper, and lead were below their respective MDLs. Thus, none of the five selected elements yielded a quantitative result at or above its respective LOQ. Exact concentrations and differences in relative abundance among the elements could not be established.

### 3.5. Effects of W. globosa Extract on Cancer Cell Viability

The effects of *W. globosa* extract on the viability of HepG2, A549, and Caco-2 cells are presented in Table 6. Cell viability remained close to that of the untreated control at the lower concentrations tested, whereas substantial reductions were observed only at the highest concentration. The estimated IC_50_ values were 41.93 ± 4.98 mg DW-equivalent/mL for HepG2 cells, 38.03 ± 4.74 mg DW-equivalent/mL for A549 cells, and 38.49 ± 1.53 mg DW-equivalent/mL for Caco-2 cells. These high IC_50_ values indicate weak inhibitory effects on cell viability in vitro under the conditions employed.

### 3.6. Effect of W. globosa Extract on IL-6 Production in H_2_O_2_-Stimulated RAW264.7 Macrophages

**Figure 2:**
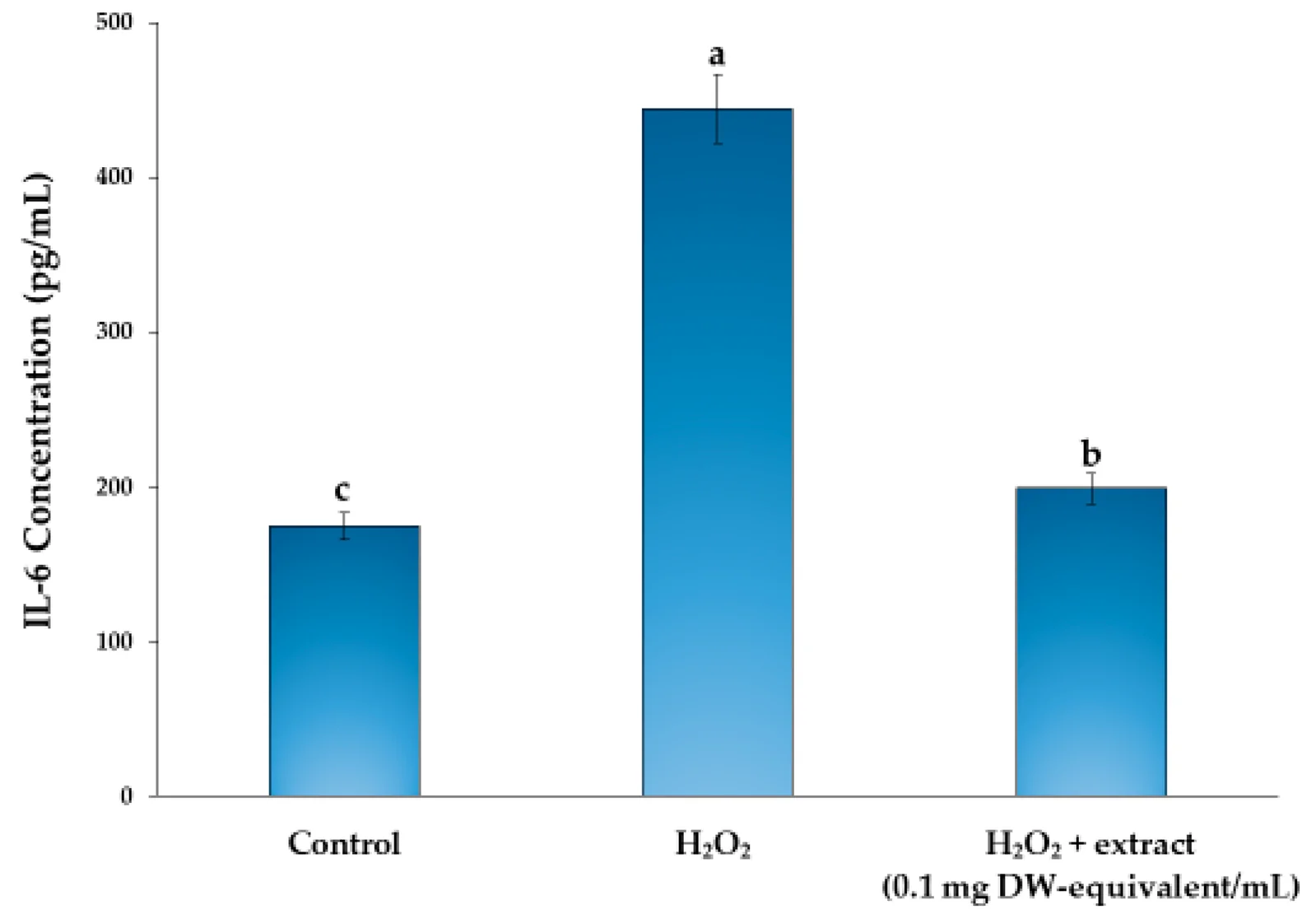
Effect of *W. globosa* extract on IL-6 production in H_2_O_2_-stimulated RAW264.7 macrophages. Cells were assigned to an untreated control group, an H_2_O_2_-stimulated extract-free model control group, and an H_2_O_2_-stimulated group treated with *W. globosa* extract at 0.1 mg DW-equivalent/mL. Bars represent the mean ± standard deviation of three technical assay replicates (*n* = 3) from a single extraction batch. Different letters indicate significant within-assay differences among groups according to one-way ANOVA followed by Duncan’s multiple range test (*p* < 0.05).

The effect of *W. globosa* extract on IL-6 production was evaluated in H_2_O_2_-stimulated RAW264.7 macrophages. As illustrated in Figure 2, the IL-6 concentration in the culture supernatant of untreated control cells was 174.43 ± 3.21 pg/mL. Exposure to H_2_O_2_ was associated with an IL-6 concentration of 444.37 ± 34.48 pg/mL, which was significantly higher than that of the untreated control (*p* < 0.05). The H_2_O_2_-stimulated group treated with *W. globosa* extract at 0.1 mg DW-equivalent/mL had an IL-6 concentration of 199.29 ± 4.16 pg/mL. This value was significantly lower than that of the H_2_O_2_-stimulated extract-free model control but remained significantly higher than that of the untreated control (*p* < 0.05).

### 3.7. Effect of W. globosa Extract on RAW264.7 Cell Viability

**Figure 3:**
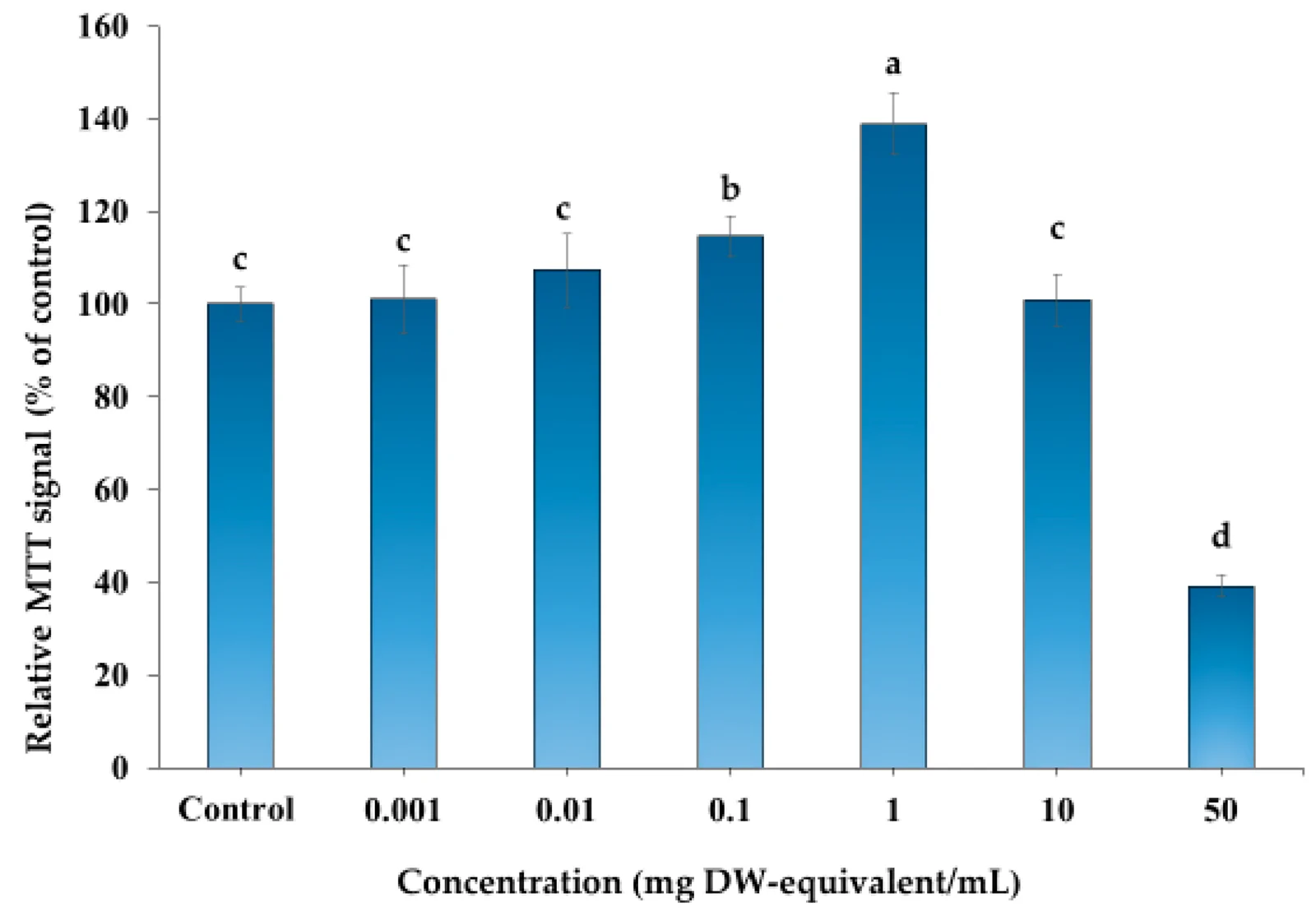
Effect of *W. globosa* extract on the MTT-derived viability of RAW264.7 macrophages. Cells were treated with different concentrations of the extract (0.001–50 mg DW-equivalent/mL) for 24 h, and relative cell viability was assessed using the MTT assay. Bars represent the mean ± standard deviation of three technical assay replicates (*n* = 3) from a single extraction batch. Different letters indicate significant within-assay differences among groups according to one-way ANOVA followed by Duncan’s multiple range test (*p* < 0.05).

The effects of *W. globosa* extract on RAW264.7 macrophage viability were assessed using the MTT assay, and the results are shown in Figure 3. At concentrations of 0.001–0.1 mg DW-equivalent/mL, the MTT-derived values remained at or above the untreated-control level. The highest value was observed at 1 mg DW-equivalent/mL, reaching 138.86 ± 6.72%, which was significantly higher than that of the untreated control (*p* < 0.05). At 10 mg DW-equivalent/mL, the MTT-derived value returned to approximately the untreated-control level. At 50 mg DW-equivalent/mL, the value decreased to 39.19 ± 2.36%, which was significantly lower than that of the other treatment groups (*p* < 0.05). The response across the tested concentrations was non-monotonic; therefore, an IC_50_ value was not reported for RAW264.7 cells.

## 4. Discussion

### 4.1. Interpretation of Nutritional Composition

The protein content of the tested *W. globosa* material was close to the previously reported values of approximately 26–27 g/100 g dry weight for this species [1]. This agreement indicates that the protein level of the present batch falls within the range described in earlier work. However, similarity in protein content does not imply that the complete proximate profiles are directly comparable, because carbohydrate, crude fiber, fat, and ash values may be influenced by differences in cultivation conditions, sample preparation, analytical definitions, and reporting basis.

In the present study, the material was washed, boiled, frozen, and freeze-dried before most compositional analyses. Published studies may use different post-harvest treatments, including analysis of unboiled, oven-dried, or otherwise processed biomass [1,3]. Boiling may result in the loss of water-soluble components, whereas drying concentrates the remaining solids. Differences in nutrient availability in the cultivation water, maturity at harvest, and other growth conditions may also contribute to differences among studies. Because the present material was obtained from a single cultivation source and a single pooled batch, the relative contributions of cultivation and processing factors cannot be distinguished.

The high moisture content and water activity of the fresh material indicate that preservation would be required for storage. Reporting the proximate composition on a dry-weight basis is therefore useful for evaluating the concentrated nutrient composition of the biomass, but it does not by itself demonstrate suitability for a particular food application. Product-specific evaluations of formulation, processing, storage stability, and microbiological quality would be required before practical use could be established.

### 4.2. Physicochemical Characteristics

The color measurements objectively describe the dark green–yellow appearance of the processed biomass. Although such a color pattern is compatible with the presence of plant pigments reported in duckweed species [3], the color coordinates cannot be used to identify or quantify chlorophylls, carotenoids, or other individual pigments. The observed color may also have been influenced by boiling, freeze-drying, grinding, and storage. Consequently, a direct comparison with published color values would require comparable sample processing and measurement conditions.

The measured pH indicates a slightly acidic to near-neutral aqueous dispersion. This value may be relevant when the material is incorporated into a food formulation, but pH alone does not determine product stability or preservation requirements, particularly given that the fresh material had high water activity. The effects of ingredient concentration, other formulation components, heat treatment, and storage conditions would need to be evaluated in the final food system.

The fat-binding capacity indicates that the powder retained oil under the conditions used in the centrifugation assay. This property may be influenced by particle size and the relative contributions of proteins and structural carbohydrate fractions [7,16]. It may therefore be relevant to the behavior of ingredients in lipid-containing formulations. However, the present assay did not measure emulsifying performance, sensory effects, lipid bioaccessibility, or gastrointestinal fat absorption; such outcomes should not be inferred directly from the FBC value.

### 4.3. Interpretation of Total Phenolic and Total Flavonoid Contents and DPPH Radical-Scavenging Activity

The measured total phenolic content and total flavonoid content are aggregate colorimetric indices expressed as gallic acid equivalents and quercetin equivalents, respectively. These measurements do not provide a chromatographic profile or establish the identities and concentrations of individual constituents. Therefore, the measured TPC and TFC values should not be interpreted as evidence for the presence, concentration, or biological activity of specific bioactive compounds. Consequently, the DPPH, IL-6, and cell-viability responses observed in this study cannot be attributed to phenolic compounds, flavonoids, or any other specific constituent or compound class. Chromatographic profiling using techniques such as HPLC or LC–MS would be required to identify individual compounds and investigate their possible relationships with the observed responses.

The extract showed weak radical-scavenging activity in the DPPH assay, with an IC_50_ value of 7.58 ± 0.32 mg DW-equivalent/mL. This relatively high IC_50_ indicates that a comparatively high concentration of the tested extract was required to reduce the DPPH radical signal by 50% under the assay conditions. The result should therefore be interpreted as preliminary evidence of activity toward the DPPH radical rather than as a comprehensive measure of antioxidant capacity. Antioxidant assays differ in their reaction mechanisms, radical systems, solvent environments, and sensitivities to individual compounds; consequently, results from a single assay cannot fully characterize the antioxidant behavior of a complex plant extract [17]. The present findings do not establish antioxidant effects in biological systems, and additional assays using complementary reaction mechanisms would be required for a broader evaluation.

### 4.4. Interpretation of Selected Elemental Levels and Reference Comparison

The findings indicate low levels of the five selected elements in the tested *W. globosa* sample. Arsenic and zinc were detected below their respective LOQs, whereas cadmium, copper, and lead were below their respective MDLs. When interpreted together with the analytical blanks, quality-control reference standard, and recoveries of 76–109%, these results are consistent with elemental levels below the method’s reliable detection or quantification ranges. Because none of the elements yielded a quantitative result at or above its respective LOQ, precise concentrations and differences in relative abundance could not be established.

The reported bounds for arsenic (<0.050 mg/kg DW), cadmium (<0.010 mg/kg DW), copper (<0.100 mg/kg DW), zinc (<0.500 mg/kg DW), and lead (<0.040 mg/kg DW) were substantially below the corresponding EFSA specification values of <1, <0.6, <8.6, <200, and <1.5 mg/kg, respectively [18]. Accordingly, none of the five selected elements exceeded the corresponding cited specification value in the tested sample.

These findings apply only to the five selected elements in the pooled sample batch examined and do not constitute a comprehensive food-safety assessment. Elemental accumulation in aquatic plants may vary with cultivation water quality, environmental conditions, harvest period, and production practices [19]. Independent sampling across cultivation sources and production batches, together with broader contaminant analysis, would therefore be required before general conclusions regarding food safety could be made.

### 4.5. Interpretation of the Effects on Cancer Cell Viability

The extract reduced the viability of HepG2, A549, and Caco-2 cells primarily at the highest concentration tested. The estimated IC_50_ values ranged from approximately 38 to 42 mg DW-equivalent/mL, indicating that comparatively high nominal starting-material-equivalent concentrations were required to reduce MTT-derived cell viability by 50%. The observed effects should therefore be described as weak inhibitory effects on cell viability in vitro rather than as evidence of anticancer activity. The high concentrations required also limit the physiological relevance of these results.

Small increases in MTT-derived values at some lower concentrations should not be interpreted as evidence of increased cell proliferation. The MTT assay reflects cellular metabolic activity and was used here as an indirect measure of cell viability. The present experiment did not include assays of apoptosis, cell-cycle regulation, or other mechanisms of cell death; therefore, the mechanism underlying the reduction in MTT signal cannot be determined from the current data.

In addition, only cancer-derived cell lines were evaluated. Without a non-cancerous comparator, it is not possible to determine whether the observed response was selective for cancer cells or represented a non-selective effect occurring at high extract concentrations. Accordingly, the present findings provide only preliminary cell-viability screening data and cannot be used to draw conclusions regarding anticancer efficacy, selectivity, or cellular safety. Future studies would require a standardized extract, appropriate noncancerous cell models, and additional mechanistic endpoints before such interpretations could be considered.

### 4.6. Effect on IL-6 Production in H_2_O_2_-Stimulated RAW264.7 Macrophages

The H_2_O_2_ model was used to examine IL-6 production following a defined oxidative-stress challenge rather than as a direct equivalent of the more conventional LPS-induced inflammatory model [15]. Accordingly, the present finding should be interpreted within the context of an oxidative-stress-associated macrophage response. Treatment with *W. globosa* extract at 0.1 mg DW-equivalent/mL was associated with a lower IL-6 concentration in H_2_O_2_-stimulated RAW264.7 culture supernatants. The H_2_O_2_-containing medium was removed after 30 min of exposure, and the cells were then treated with the extract. The observation therefore reflects the cellular response following a limited H_2_O_2_ exposure rather than continuous co-exposure to H_2_O_2_ and the extract. Nevertheless, only one extract concentration and one inflammatory marker were evaluated. The finding does not establish a dose-dependent response, a broad anti-inflammatory effect, or a specific mechanism of action.

The experimental design included an untreated control and an H_2_O_2_-stimulated extract-free model control. The H_2_O_2_-stimulated group served as the model control and should not be interpreted as a pharmacological positive anti-inflammatory control. A concentration-matched vehicle control, an extract-only group within the IL-6 experiment, a recognized anti-inflammatory reference compound, and a dose–response series were not included. Moreover, the separate RAW264.7 MTT experiment evaluated cells treated with the extract alone, whereas cell viability was not assessed under the combined H_2_O_2_-plus-extract condition. Consequently, the possibility that the lower IL-6 concentration was influenced by altered cell number or metabolic activity under the combined treatment cannot be excluded.

Residual ethanol in the post-evaporation concentrate was not analytically quantified, and a concentration-matched ethanol vehicle control was not included. Therefore, a possible contribution from unmeasured residual solvent cannot be completely excluded. Accordingly, the lower IL-6 concentration cannot be attributed specifically to an anti-inflammatory effect of the extract under the present experimental design. Taken together, these limitations require that the finding be interpreted as a preliminary, assay-specific observation regarding IL-6 production rather than as confirmation of anti-inflammatory efficacy.

Future studies should use a dried, weighed, and standardized extract together with a matched vehicle control, an extract-only group, an appropriate pharmacological positive control, viability assessment under the combined stimulation-plus-extract condition, and multiple extract concentrations. Additional inflammatory endpoints, including TNF-α, IL-1β, nitric oxide production, iNOS expression, and COX-2 expression, would provide a broader evaluation of the inflammatory response [4,20].

### 4.7. Interpretation of RAW264.7 Cell-Viability Responses

The RAW264.7 MTT results showed a non-monotonic response, with MTT-derived values remaining at or above the control level at lower and intermediate extract concentrations and declining markedly at the highest concentration tested. Because the MTT assay measures cellular reductive metabolic activity rather than cell number directly, an increased MTT signal should not be interpreted as evidence of cell proliferation [14]. Likewise, the reduced signal at the highest concentration is consistent with decreased metabolic activity and/or viability but does not identify the underlying mechanism.

These findings are therefore useful primarily for describing the response of RAW264.7 cells across the tested concentrations under the assay conditions and for identifying concentrations that did not produce an overt reduction in the MTT signal. They do not demonstrate a stimulatory effect at low concentrations or a specific pathway of cell injury at high concentrations. In addition, because the viability and IL-6 experiments were conducted as separate assays, the viability findings provide contextual information for concentration selection but do not establish the mechanism underlying the observed change in IL-6 production.

### 4.8. Limitations and Future Perspectives

Several limitations should be acknowledged. First, antioxidant evaluation was limited to the DPPH radical-scavenging assay; therefore, the result reflects only activity toward the DPPH radical under the conditions employed and does not constitute a comprehensive assessment of antioxidant capacity. Additional assays based on complementary reaction mechanisms would be required for broader characterization [17].

Second, the IL-6 experiment evaluated only one extract concentration and one inflammatory marker and lacked a concentration-matched vehicle control, an extract-only group, a pharmacological positive control, a dose–response series, and cell-viability assessment under the combined H_2_O_2_-plus-extract condition; residual ethanol was also not analytically quantified. Accordingly, the IL-6 finding remains a preliminary, assay-specific observation rather than evidence of broad anti-inflammatory efficacy.

Third, reductions in cancer-cell viability occurred only at relatively high extract concentrations. No non-cancerous comparator cells or mechanistic endpoints were included; therefore, anticancer efficacy, selectivity, and cellular safety could not be established. Likewise, the RAW264.7 MTT assay measured reductive metabolic activity and did not identify the mechanism underlying the observed responses.

Fourth, chemical characterization of the crude extract was limited to colorimetric determinations of total phenolic and total flavonoid contents, which do not identify or quantify individual compounds. Consequently, the observed DPPH, IL-6, and cell-viability responses cannot be attributed to specific constituents; compound-level characterization would require techniques such as HPLC or LC–MS.

Fifth, all analyses were based on one pooled *W. globosa* batch from a single cultivation source, and the reported triplicates were technical rather than independent biological replicates. Detailed cultivation and harvest records were unavailable, and no voucher specimen was deposited. The selected-element analysis was also limited to five elements. Consequently, the findings characterize only the tested batch, cannot be generalized across cultivation sources, harvests, or production batches, and do not constitute a comprehensive food-safety assessment. Accordingly, the reported mean ± SD values and statistical comparisons reflect technical or analytical variation within the tested pooled sample or single extraction batch and should not be interpreted as biological variation among independent *W. globosa* samples.

An additional methodological limitation concerns the standardization of extracts. The post-evaporation concentrate was not dried or weighed; therefore, treatment concentrations represent nominal starting-material dry-weight equivalents rather than dried-extract concentrations. Future studies should use dried, weighed, and standardized extracts together with appropriate vehicle controls and independent extraction batches.

Finally, all biological responses were evaluated in vitro and do not fully represent physiological or food-system complexity. Further work should include independent sample batches, food-formulation and bioaccessibility studies, and, where scientifically justified, additional biological models.

Within these limitations, the study provides coordinated data on nutritional composition, physicochemical properties, selected elements, total phenolic and total flavonoid contents, and preliminary in vitro responses derived from the same pooled *W. globosa* batch and processing sequence. This common sample context provides a defined baseline for further investigation; however, it does not support conclusions regarding compound-specific mechanisms, broad biological efficacy, comprehensive food safety, or consistency across cultivation and production conditions.

## 5. Conclusions

This study characterized the nutritional composition and physicochemical properties of a single pooled batch of *W. globosa* and evaluated selected elemental levels and preliminary in vitro responses. The freeze-dried powder from this pooled batch contained nutritionally relevant amounts of protein and crude fiber and exhibited physicochemical characteristics that may be relevant to its further evaluation as a plant-based food ingredient. The selected-element analysis indicated low levels of the five selected elements in the tested sample. Arsenic and zinc were detected below their respective LOQs, whereas cadmium, copper, and lead were below their respective MDLs. The analytical reporting limits for all five elements were below the corresponding specification values reported for *W. globosa* powder in the EFSA scientific opinion. Accordingly, none of the selected elements exceeded the cited specification value in the tested sample. However, because only five selected elements and one pooled sample batch were examined, these findings do not constitute a comprehensive assessment of food safety.

The extract showed measurable responses in the total phenolic and total flavonoid assays but weak DPPH radical-scavenging activity under the assay conditions. These aggregate colorimetric measurements do not identify individual phytochemical compounds. The H_2_O_2_-stimulated group treated with the extract at 0.1 mg DW-equivalent/mL showed a lower IL-6 concentration than the H_2_O_2_-stimulated extract-free model control. However, only one extract concentration was evaluated, no extract-only or concentration-matched vehicle control was included, and cell viability was not assessed under the combined H_2_O_2_-plus-extract condition. Therefore, this finding should be interpreted only as a preliminary, assay-specific observation regarding IL-6 production and not as evidence of anti-inflammatory efficacy. Weak inhibitory effects on cell viability in HepG2, A549, and Caco-2 cells were observed only at high extract concentrations, with IC_50_ values of approximately 38–42 mg DW-equivalent/mL. These findings do not support claims of anticancer potential, and the absence of a non-cancerous cell line prevents conclusions regarding selectivity or safety.

Overall, within the scope of this single pooled batch, the tested *W. globosa* material showed promising nutritional and physicochemical characteristics, low levels of selected elements, and preliminary in vitro responses. Sustainability was not directly evaluated using biomass productivity, resource use, environmental impact assessment, or life-cycle analysis, and comprehensive food safety should not be inferred from the present results. Further studies using independent cultivation and harvest batches, broader contaminant analyses, standardized extracts, food-formulation models, bioaccessibility and bioavailability assessments, and more comprehensive biological experiments are required.

## Figures and Tables

**Table 1:** Proximate composition of freeze-dried *W. globosa* powder on a dry-weight basis.

Nutrient Composition	Content (DW)
Energy (kcal/100 g)	321.24 ± 1.63
Protein (%)	25.83 ± 2.65
Fat (%)	4.56 ± 0.15
Ash (%)	16.58 ± 0.35
Crude fiber (%)	8.80 ± 0.61
Carbohydrate (%)	44.22 ± 2.48

Values are expressed as mean ± standard deviation of three technical replicate measurements (*n* = 3) from the same pooled sample batch. The analyzed powder was obtained from plant material boiled before freeze-drying. DW = dry weight.

**Table 2:** Moisture content and water activity of fresh *W. globosa*.

Parameter	Value (Mean ± SD)
Moisture (%)	96.78 ± 0.07
Water activity (a_w_)	0.96 ± 0.01

Values are expressed as mean ± standard deviation of three technical replicate measurements (*n* = 3) from the same pooled sample batch. Measurements were performed after washing and before boiling. Moisture content is expressed on a fresh-weight basis (FW).

**Table 3:** Physicochemical properties of freeze-dried *W. globosa* powder.

Physicochemical Properties	Value (Mean ± SD)
L*	30.43 ± 0.69
a*	−7.13 ± 0.25
b*	+33.09 ± 1.09
pH	6.13 ± 0.57
FBC (mL oil/g sample)	1.92 ± 0.15

Values are expressed as mean ± standard deviation of three technical replicate measurements (*n* = 3) from the same pooled sample batch. L* = lightness; a* = redness/greenness; b* = yellowness/blueness.

**Table 4:** Total phenolic and total flavonoid contents and DPPH radical-scavenging activity of *W. globosa* extract.

Parameter	Value (Mean ± SD)
Total phenolic content (mg GAE/g DW of sample)	0.85 ± 0.03
Total flavonoid content (mg QE/g DW of sample)	0.52 ± 0.02
DPPH scavenging activity (IC_50_, mg DW-equivalent/mL)	7.58 ± 0.32

Values are expressed as mean ± standard deviation of three technical assay replicates (*n* = 3) from a single extraction batch. GAE = gallic acid equivalents; QE = quercetin equivalents; DW = dry weight of the original *W. globosa* sample; IC_50_ = concentration required to inhibit 50% of DPPH radicals. DW-equivalent indicates the equivalent mass of the starting freeze-dried *W. globosa* material and does not represent the mass of dried extract solids.

**Table 5:** Levels of selected elements in freeze-dried *W. globosa* powder.

Element	Analytical Result (mg/kg DW)	MDL (mg/kg DW)	LOQ (mg/kg DW)
Arsenic (As)	Detected, <0.050	0.010	0.050
Cadmium (Cd)	<0.010	0.010	0.050
Copper (Cu)	<0.100	0.100	0.500
Zinc (Zn)	Detected, <0.500	0.320	0.500
Lead (Pb)	<0.040	0.040	0.100

MDL = method detection limit; LOQ = limit of quantification; DW = dry weight. Signals at or above the MDL but below the LOQ were reported as detected, <LOQ; results below the MDL were reported as <MDL. Method recoveries ranged from 76% to 109% across the analyzed elements. Analyses were based on three technical replicate measurements from the same pooled sample batch.

**Table 6:** Effects of *W. globosa* extract on the viability of HepG2, A549, and Caco-2 cells.

Concentration (mg DW-Equivalent/mL)	HepG2 Cell Viability (%)	A549 Cell Viability (%)	Caco-2 Cell Viability (%)
Ctrl	100.00 ± 8.08 ^b^	100.00 ± 4.99 ^a^	100.00 ± 4.61 ^b^
0.001	109.88 ± 10.85 ^ab^	100.10 ± 2.04 ^a^	102.63 ± 5.71 ^b^
0.01	121.61 ± 9.67 ^a^	100.57 ± 9.08 ^a^	116.81 ± 6.08 ^a^
0.1	110.48 ± 10.84 ^ab^	100.97 ± 6.39 ^a^	107.97 ± 3.87 ^b^
1	103.15 ± 8.43 ^b^	92.05 ± 9.18 ^a^	101.66 ± 3.94 ^b^
10	93.39 ± 8.36 ^b^	76.68 ± 4.37 ^a^	85.64 ± 5.01 ^c^
50	39.10 ± 6.78 ^c^	38.59 ± 4.58 ^c^	35.55 ± 3.02 ^d^
IC_50_ value (mg DW-equivalent/mL)	41.93 ± 4.98	38.03 ± 4.74	38.49 ± 1.53

Values are expressed as mean ± standard deviation of three technical assay replicates (*n* = 3) from a single extraction batch. Different superscript letters within the same column indicate significant differences at *p* < 0.05. IC_50_ represents the concentration of extract required to reduce cell viability by 50% and was calculated from dose–response curves. DW-equivalent indicates the equivalent mass of the starting freeze-dried *W. globosa* material and does not represent the mass of dried extract solids.

## Data Availability

The data presented in this study are available on request from the corresponding author.
